# Nucleocapsid protein of SARS-CoV-2 phase separates into RNA-rich polymerase-containing condensates

**DOI:** 10.1038/s41467-020-19843-1

**Published:** 2020-11-27

**Authors:** Adriana Savastano, Alain Ibáñez de Opakua, Marija Rankovic, Markus Zweckstetter

**Affiliations:** 1 German Center for Neurodegenerative Diseases (DZNE), Von-Siebold-Str. 3a, 37075 Göttingen, Germany; 2 Department for NMR-based Structural Biology, Max Planck Institute for Biophysical Chemistry, Am Faßberg 11, 37077 Göttingen, Germany

**Keywords:** Structural biology, Intrinsically disordered proteins, Solution-state NMR

## Abstract

The etiologic agent of the Covid-19 pandemic is the severe acute respiratory syndrome coronavirus 2 (SARS-CoV-2). The viral membrane of SARS-CoV-2 surrounds a helical nucleocapsid in which the viral genome is encapsulated by the nucleocapsid protein. The nucleocapsid protein of SARS-CoV-2 is produced at high levels within infected cells, enhances the efficiency of viral RNA transcription, and is essential for viral replication. Here, we show that RNA induces cooperative liquid–liquid phase separation of the SARS-CoV-2 nucleocapsid protein. In agreement with its ability to phase separate in vitro, we show that the protein associates in cells with stress granules, cytoplasmic RNA/protein granules that form through liquid-liquid phase separation and are modulated by viruses to maximize replication efficiency. Liquid–liquid phase separation generates high-density protein/RNA condensates that recruit the RNA-dependent RNA polymerase complex of SARS-CoV-2 providing a mechanism for efficient transcription of viral RNA. Inhibition of RNA-induced phase separation of the nucleocapsid protein by small molecules or biologics thus can interfere with a key step in the SARS-CoV-2 replication cycle.

## Abstract (web summary)

The SARS-CoV-2 viral genome is encapsulated by the nucleocapsid protein (N^SARS-CoV-2^) that is essential for viral replication. Here, the authors show that RNA induces liquid-liquid phase separation of N^SARS-CoV-2^ and how N^SARS-CoV-2^ phosphorylation modulates RNA-binding and phase separation and that these RNA/N^SARS-CoV-2^-droplets recruit and concentrate the SARS-CoV-2 RNA-dependent RNA polymerase complex in vitro, which would enable high initiation and elongation rates during viral transcription.

## Introduction

The etiologic agent of the Covid-19 pandemic is the severe acute respiratory syndrome coronavirus 2 (SARS-CoV-2) (https://www.who.int/emergencies/diseases/novel-coronavirus-2019). SARS-CoV-2 is an enveloped single-stranded, positive-sense RNA virus with a 30 kb genome, one of the largest among RNA viruses^1,2^. The viral membrane of SARS-CoV-2, which contains the spike protein, a glycoprotein, and the envelope protein^1,3^, surrounds a helical nucleocapsid. In the nucleocapsid, the viral genome is encapsulated by the nucleocapsid protein and thereby protected from the host cell environment^4,5^. The nucleocapsid protein of human coronaviruses is produced at high levels within infected cells and is critical for virion assembly^4–7^. In addition, it enhances the efficiency of sub-genomic viral RNA transcription and is essential for viral replication^4^. Because of its importance for diagnostic and therapeutic approaches to treat Covid-19^8,9^, there is an urgent need to define the molecular mechanisms that underlie the nucleocapsid protein’s fundamental viral role.

Liquid–liquid phase separation (LLPS) provides a highly cooperative mechanism to locally concentrate proteins and nucleic acids and promote cellular reactions^10,11^. Recent evidence indicates that negative-sense RNA viruses, which replicate in the cytoplasm of infected cells^12^, concentrate their replication machinery in dynamic compartments formed by LLPS of the viral structural proteins L, phosphoprotein (P), and nucleocapsid (N) protein^13,14^. The genomes of positive-sense RNA viruses such as SARS-CoV-2, however, lack the genetic code for the phosphoprotein P, which is essential for LLPS in negative-sense RNA viruses^13–15^.

Here we investigate liquid–liquid phase separation of the nucleocapsid protein of SARS-CoV-2 and show that nucleocapsid protein LLPS concentrates components of the SARS-CoV-2 replication machinery providing a mechanism for enhanced viral transcription and replication.

## Results

### LLPS of N^SARS-CoV-2^ and RNA into protein/RNA-dense compartments

**Fig. 1:**
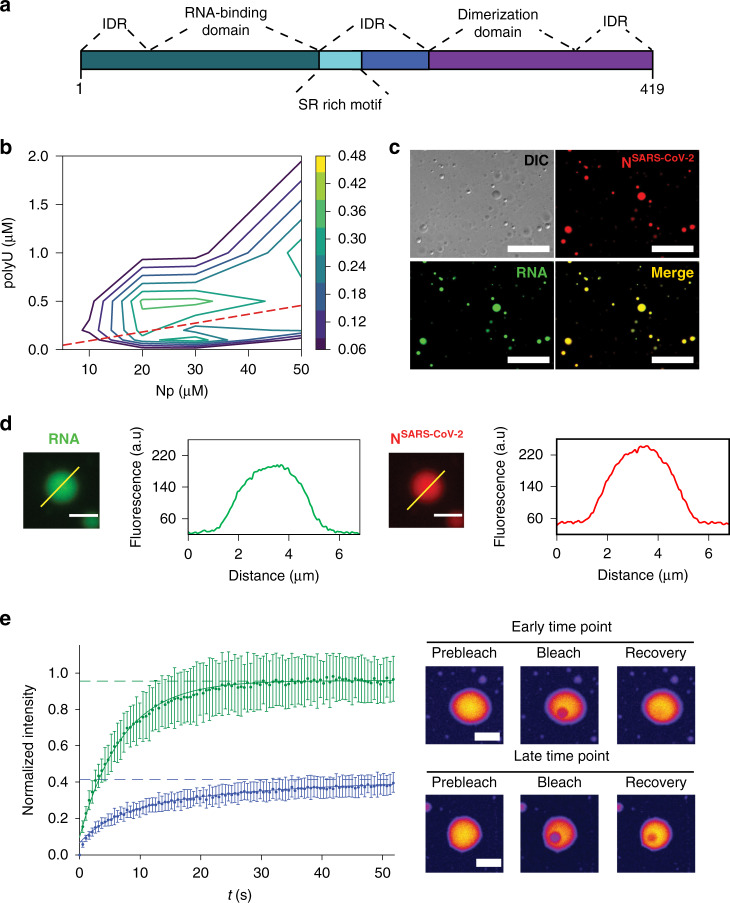
RNA-induced LLPS of the nucleocapsid protein of SARS-CoV-2. **a** Organization of N^SARS-CoV-2^ into two globular domains (RNA-binding domain and C-terminal dimerization domain) surrounded by long intrinsically disordered regions (IDR)^4,5^. The serine/arginine (SR)-rich region is conserved in coronaviruses. **b** Influence of RNA and protein concentration on N^SARS-CoV-2^/polyU-LLPS in 20 mM NaPi, pH 7.5, monitored by solution turbidity at 350 nm. Average values from three independent measurements are shown. The dashed line marks N^SARS-CoV-2^/polyU-concentrations at which charge neutralization occurs, assuming a charge of −1 per phosphate group. **c** Fluorescence and DIC microscopy of spherical droplets of 50 µM N^SARS-CoV-2^ and 1 µM polyU in 20 mM NaPi, pH 7.5. Fluorescently labeled RNA (green) partitioned into the droplets. Scale bar, 20 µm. Micrographs are representative of three independent biological replicates. **d** Increase in N^SARS-CoV-2^- and RNA-concentration inside of N^SARS-CoV-2^/polyU droplets in 20 mM NaPi, pH 7.5. Scale bars 3 µm. Micrographs are representative of three independent biological replicates. **e** Time-dependent change in diffusion of N^SARS-CoV-2^ inside polyU-induced droplets observed by FRAP. FRAP of freshly prepared droplets is shown in green, and after incubation for one hour in blue. Error bars represent the standard deviation for averaged six curves for each time point. Representative micrographs of a fresh droplet (top) and after incubation for one hour (bottom) before bleaching, after bleaching, and at the end of recovery are displayed to the right. Scale bars 10 µm.

To investigate if the N protein of SARS-CoV-2 (further termed N^SARS-CoV-2^; Fig. 1a) phase separates in the absence of other viral proteins, we measured the turbidity of N^SARS-CoV-2^ solutions at different protein concentrations. Up to 50 µM, the protein solution retained low absorbance (Fig. 1b), despite its tendency to oligomerize^16^. Next, we tested LLPS of N^SARS-CoV-2^ in the presence of RNA. The 419-residue N^SARS-CoV-2^ contains an RNA-binding domain and a C-terminal dimerization domain embedded into long intrinsically disordered regions (Fig. 1a)^4,5^. The globular domains as well as the intrinsically disordered regions of coronavirus N proteins bind to RNA^4^. At 50 µM protein concentration, the addition of 1 µM polyU (800 kDa), which we used as a substitute for viral RNA, strongly increased turbidity (Fig. 1b). Differential interference contrast (DIC) and fluorescent microscopy demonstrated the formation of spherical droplets (Fig. 1c). The droplets contained both N^SARS-CoV-2^ and RNA (Fig. 1c). N^SARS-CoV-2^/polyU droplets were robust against the presence of the aliphatic alcohol 1,6-hexanediol (Supplementary Fig. 1a). In contrast, the addition of increasing amounts of NaCl dissolved the droplets (Supplementary Fig. 1b), indicating an important role of electrostatic interactions for RNA-induced LLPS of N^SARS-CoV-2^. Quantification of fluorescence intensities of N^SARS-CoV-2^ and RNA showed that their concentration is strongly increased inside droplets (Fig. 1d), i.e., cooperative LLPS of N^SARS-CoV-2^ and RNA into protein/RNA-dense compartments occurs.

Next, we monitored LLPS for different N^SARS-CoV-2^ and polyU concentrations. According to turbidity measurements, polyU-induced LLPS started at 5–10 µM N^SARS-CoV-2^ (Fig. 1b and Supplementary Fig. 1c). The polyU concentration at which maximum turbidity was observed shifted to higher polyU concentrations with increasing protein concentration (Fig. 1b and Supplementary Fig. 1c). Calculation of the N^SARS-CoV-2^ and polyU charge showed that LLPS is strongest when charge neutralization occurs (Fig. 1b). At a given protein concentration, the turbidity increased with increasing polyU concentration, reached a maximum, and then rapidly decreased to its starting value (Fig. 1b), i.e., charge-matching RNA concentrations enable phase separation, but high RNA/protein ratios prevent LLPS of N^SARS-CoV-2^. The RNA-induced LLPS behavior of N^SARS-CoV-2^ is in agreement with the known properties of RNA-induced phase separation of prion-like RNA binding proteins^17^.

### Time-dependent transformation of N^SARS-CoV-2^/RNA-droplets

A characteristic property of LLPS is the liquid-like nature of phase-separated compartments. We photobleached N^SARS-CoV-2^ inside of N^SARS-CoV-2^/polyU droplets and observed rapid recovery of fluorescence (Fig. 1e). We then waited one hour and repeated fluorescence recovery after photobleaching (FRAP). At this later time point, the fluorescence recovery was best described by a bi-exponential fit consisting of two components (Fig. 1e and Supplementary Fig. 1d). In addition, the fluorescence did not fully recover (Fig. 1e), i.e., ~60% of N^SARS-CoV-2^ had transformed into an immobile species. The analysis shows that N^SARS-CoV-2^/polyU droplets change their material properties in a time-dependent manner. Because a major activity of N^SARS-CoV-2^ is to encapsulate RNA, the immobile fraction observed by FRAP might represent the early stages of nucleocapsid assembly. Successful nucleocapsid formation, however, also depends on the specific sequence and secondary structure of viral RNA^18^ and is therefore not expected for polyU.

### SARS-CoV-2 nucleocapsid protein associates with stress granules

**Fig. 2:**
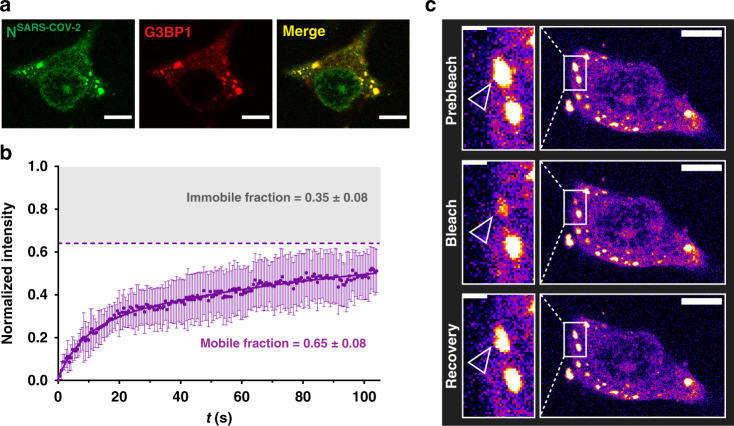
Nucleocapsid protein of SARS-CoV-2 associates with stress granules. **a** Alexa Fluor 488 labeled N^SARS-CoV-2^ (green) colocalizes with the stress granule marker G3BP1 (red) in arsenite-treated digitonin-permeabilized HeLa cells. Micrographs are representative of three independent biological replicates. **b** Fluorescence recovery after photobleaching (FRAP) curve fitted with bi-exponential fit suggests the existence of a fast diffusing population of molecules, which together with a slowly recovering population comprises ~65% of the bleached spot (mobile fraction). The remaining 35% are immobile and do not recover its fluorescence after photobleaching. The curve is the average of *n* = 6 stress granules, error bars represent standard deviation. **c** Corresponding confocal microscopy pictures of the representative FRAP of N^SARS-CoV-2^ associated with stress granules in HeLa cells. Insert shows the bleached stress granule marked with an arrow. Micrographs are representative of *n* = 6 FRAP experiments in one biological sample. Scale bar 10 µm in (**a**) and (**c**), 2 µm in the inset in (**c**).

Stress granules (SGs) are cytoplasmic RNA/protein granules, which form through LLPS and are modulated by corona- and other viruses to maximize replication efficiency^19,20^. SARS-CoV-2 protein interaction mapping indicated that N^SARS-CoV-2^ binds the SG protein G3BP1^21^. To investigate if N^SARS-CoV-2^ associates with SGs, we used a previously established SG-colocalization assay^22^. SGs were induced in HeLa cells by arsenite followed by permeabilization of the plasma membrane by digitonin. Subsequently, soluble cytosolic factors were washed out and fluorescently labeled N^SARS-CoV-2^ was added together with Alexa Fluor 594-coupled antibody against the SG marker G3BP1. Laser scanning confocal microscopy showed arsenite-induced formation of SGs that stained for G3BP1 (Fig. 2a). N^SARS-CoV-2^ colocalized with G3BP1-positive SGs (Fig. 2a; Supplementary Movie 1). FRAP of SG-associated N^SARS-CoV-2^ suggested the presence of three N^SARS-CoV-2^ populations (Fig. 2b): a very mobile with rapid fluorescence recovery, a slower diffusing component, and an immobile fraction, which does not recover its fluorescence after photobleaching (Fig. 2c). Because SGs consists of a rigid core and a dynamic shell^23^, we attribute the different N^SARS-CoV-2^ diffusion properties to the localization of N^SARS-CoV-2^ to different sub-structures of SGs. In agreement with our findings for N^SARS-CoV-2^, the N protein of SARS-CoV, the causative agent of the SARS epidemic in 2002/2003, translocates to SGs in stressed SARS-CoV-infected cells^24^.

### RNA-interaction of the mutation-prone SR-region

**Fig. 3:**
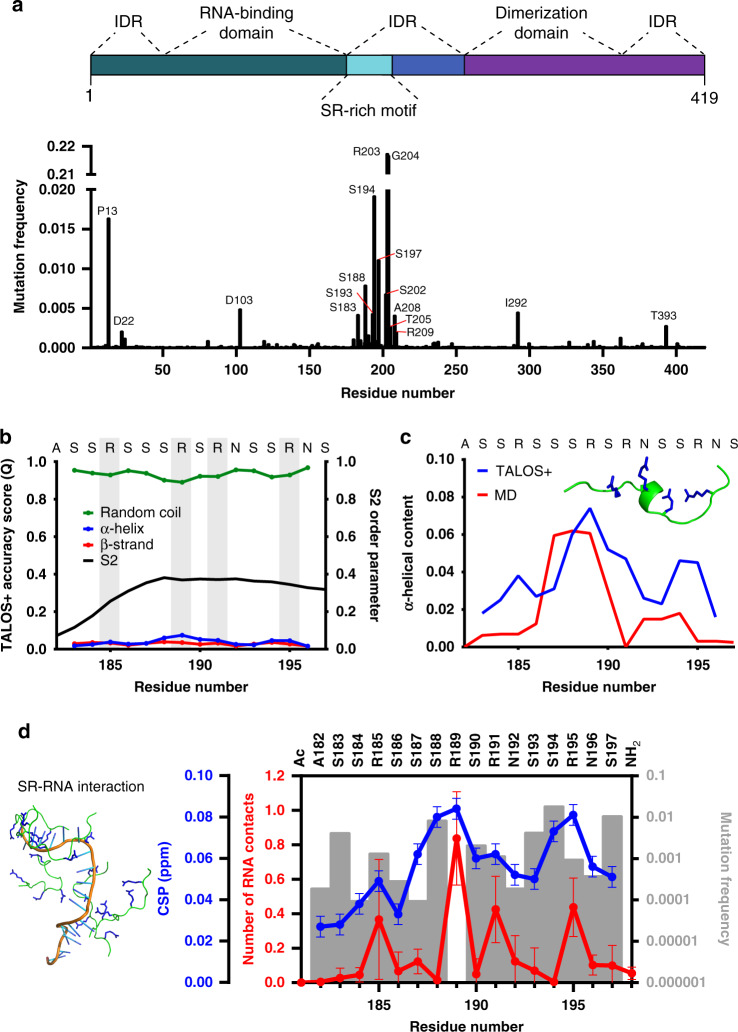
Atomic details of the RNA-interaction of the mutation-prone SR-region. **a** Frequency of mutations in the nucleocapsid protein in 42176 SARS-CoV-2 sequences from the China National Center for Bioinformation. Residues with more than 0.0025 frequency are labeled. Domain organization of N^SARS-CoV-2^ on top. **b** NMR-based analysis of the structure of the high-density SR-stretch (residues A182-S197) of N^SARS-CoV-2^. Secondary structure derived from chemical shifts using TALOS+^36^ is represented together with the S^2^ order parameter. Arginines are highlighted in gray. **c** Comparison between the α-helical propensity derived from NMR data (blue) and MD simulations (red). One conformer with α-helical content from the simulation is shown inside the graph. **d** Comparison between the number of peptide-polyU contacts per peptide in the MD simulations (red; average and standard deviation of five simulations), the NMR chemical shift perturbation (CSP) of the peptide-polyU titration at 1500 nM of polyU (blue), and the frequency of mutations per residue (gray bars). At the left, a MD snapshot of five SR-peptides with the 20 base length polyU is shown.

RNA viruses have enormously high mutation rates enhancing virulence and evolvability. On June 7th 2020, already 42176 SARS-CoV-2 sequences were deposited (https://www.gisaid.org). Analysis of the corresponding N proteins showed that the mutations are most frequent in the SR-region of N^SARS-CoV-2^ (Fig. 3a), which is conserved among human coronaviruses (Supplementary Fig. 2). SR-regions bind both RNA and proteins^25^. To gain insight into the molecular properties of the SR-region of N^SARS-CoV-2^ and its interaction with RNA, we combined NMR spectroscopy with molecular dynamics (MD) simulations. We particularly focused on the region from A182 to S197, because it contains 9 serine and 4 arginine residues, i.e., the highest density of SR-motifs in N^SARS-CoV-2^ (Supplementary Fig. 2). Chemical shift analysis showed that residues A182-S197 are very dynamic with a small propensity of α-helical structure next to R189 (Fig. 3b-c).

Next, we investigated the conformational properties of A182-S197 with MD simulations. For the simulations, we used the state-of-the-art force field/water model that accurately reproduced NMR parameters of the intrinsically disordered protein α-synuclein^26^. In agreement with the NMR analysis, residues next to R189 populate transient α-helical structure (Fig. 3c). We then performed calculations in the presence of polyU. A large number of intermolecular contacts between the arginine residues and the RNA phosphate groups were observed. Most intermolecular contacts were present for R189 (Fig. 3d, red; Supplementary Fig. 3). In addition, R189 was most sensitive to the addition of polyU as observed by NMR spectroscopy (Fig. 3d, blue; Supplementary Fig. 4a). R189 is the only residue in the region from A182-S197 that is not mutated in 42176 SARS-CoV-2 sequences (Fig. 3d, gray bars), in agreement with its functional relevance.

### SR-phosphorylation modulates RNA-induced phase separation

**Fig. 4:**
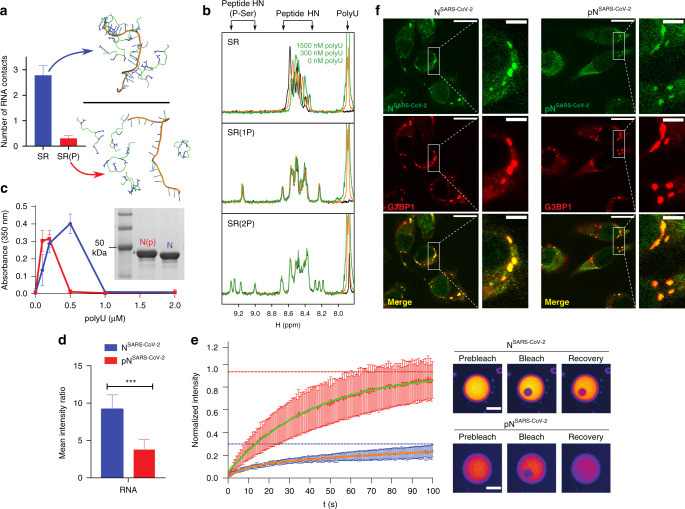
SR-phosphorylation modulates RNA-induced condensation of the nucleocapsid protein. **a** MD simulations of SR/polyU-interaction. The number of contacts per peptide with a 20 base length polyU is shown in the top graph for the non-phosphorylated and fully phosphorylated SR-peptide as the average and standard deviation of five independent simulations. Snapshots of both peptides are shown. **b** ^1^H 1D experiments of the SR-peptide (1766.8 Da), SPRK1 single-phosphorylated peptide (SR(1 P); 1846.9 Da according to mass spectrometry) and SPRK1 double-phosphorylated peptide (SR(2 P); 1926.9 Da according to mass spectrometry; Supplementary Fig. 8) at three different concentrations of polyU (0, 300 and 1500 nM). The spectral regions, in which the signals of polyU, the backbone H^N^s of unmodified residues and phosphorylated serines are located, are marked. The positive charges of the SR-peptide are compensated by the polyU negative charges at around 300 nM. **c** Turbidity at 350 nm of solutions of non-phosphorylated (blue) and SRPK1-phosphorylated (red) N^SARS-CoV-2^ in 20 mM NaPi, pH 7.5, at 30 µM protein concentration and increasing concentrations of polyU. Average values from three independent measurements are shown. Error bars, std. An SDS-Page gel (insert) displays a band shift due to SRPK1-phosphorylation. **d** Decreased RNA recruitment into polyU-induced droplets of N^SARS-CoV-2^ upon phosphorylation with SRPK1 kinase. Mean values and standard deviation are displayed (n = 100 droplets). Two-sided t test with *P* value set to < 0.05 for statistical significance, *** < 0.001, ** < 0.002 and * < 0.033, ns < 0.12. **e** FRAP of N^SARS-CoV-2^ (blue) and SRPK1-phosphorylated N^SARS-CoV-2^ (red) inside of polyU-induced droplets. Error bars represent the standard deviation for averaged 8 and 10 curves for unmodified and phosphorylated N^SARS-CoV-2^, respectively. Representative micrographs of N^SARS-CoV-2^ (top) and SRPK1-phosphorylated N^SARS-CoV-2^ droplets (bottom) before bleaching, after bleaching and at the end of recovery are displayed to the right. Partition coefficients of 12.2 ± 2.8 and 4.3 ± 1.2 were calculated for N^SARS-CoV-2^ and phosphorylated N^SARS-CoV-2^, respectively. Scale bars, 10 µm. **f** Association of unmodified (N^SARS-CoV-2^, left panels) and phosphorylated (phosphoN^SARS-CoV-2^, right panels) nucleocapsid protein of SARS-CoV-2 with stress granules in HeLa cells, micrographs are representative of two independent biological replicates. Scale bar 20 µm, 5 µm in inset.

Phosphorylation of SR-domains provides functional specificity and adjustability to ribonucleoprotein formation^25^ and impairs binding of SR-domains of pre-mRNA splicing factors to protein hydrogel droplets^27^. To gain insight into the impact of phosphorylation of the SR-region of N^SARS-CoV-2^ on its RNA binding, we performed MD simulations of the high-density SR-stretch carrying phosphate groups at different serine residues (Supplementary Fig. 5a). Even when only a single serine, S188, was phosphorylated, the number of intra- and intermolecular peptide/peptide contacts increased (Supplementary Fig. 5b-c). Multi-site phosphorylation further raised the number of contacts (Supplementary Fig. 5b–c), and the intermolecular peptide/peptide contacts reached a maximum when three serines were phosphorylated (Supplementary Fig. 5c), i.e., when the overall charge is around zero. The phosphorylation-induced increase in intra- and intermolecular contacts is predominantly due to the formation of salt bridges between the phosphate groups and arginine side chains (Supplementary Fig. 5b–c). Because of the dense network of intra- and interpeptide salt bridges, contact formation with RNA—either polyU or a structured RNA derived from the viral genome of SARS-CoV-2—was strongly attenuated upon phosphorylation (Fig. 4a and Supplementary Fig. 6).

To validate the results from MD simulation, we phosphorylated the SR-peptide in vitro using the serine/arginine protein kinase 1 (SRPK1) and performed NMR spectroscopy. SRPK1 phosphorylates SR-motifs and is involved in a wide spectrum of cellular activities including the regulation of viral genome replication^25,28^. SRPK1-phosphorylation resulted in two species, a single phosphorylation at S188 (Fig. 4b, middle) and a di-phosphorylated state, which is heterogeneously phosphorylated at four different serines (Fig. 4b, bottom; Supplementary Fig. 7). NMR titrations showed that the unmodified SR-peptide strongly interacts with polyU, but not when it is phosphorylated at S188 (Fig. 4b and Supplementary Fig. 4b). LLPS experiments further demonstrated that phosphorylation of full-length N^SARS-CoV-2^ by SRPK1 changes its RNA-induced phase separation behavior (Fig. 4c and Supplementary Fig. 8). The maximum of RNA-induced turbidity was shifted to lower polyU-concentrations for SRPK1-phosphorylated N^SARS-CoV-2^ (Fig. 4c). In addition, fluorescently labeled RNA was less recruited to droplets formed by SRPK1-phosphorylated N^SARS-CoV-2^ (Fig. 4d). In agreement with an attenuated interaction of N^SARS-CoV-2^ with RNA upon SRPK1-phosphorylation, we also observed a more rapid diffusion of SRPK1-phosphorylated N^SARS-CoV-2^ inside of polyU-induced droplets when compared to the unmodified protein (Fig. 4e). On the other hand, SRPK1-phosphorylated N^SARS-CoV-2^ still co-localized with stress granules (Fig. 4d).

### Nucleocapsid protein LLPS concentrates components of the SARS-CoV-2 replication machinery

**Fig. 5:**
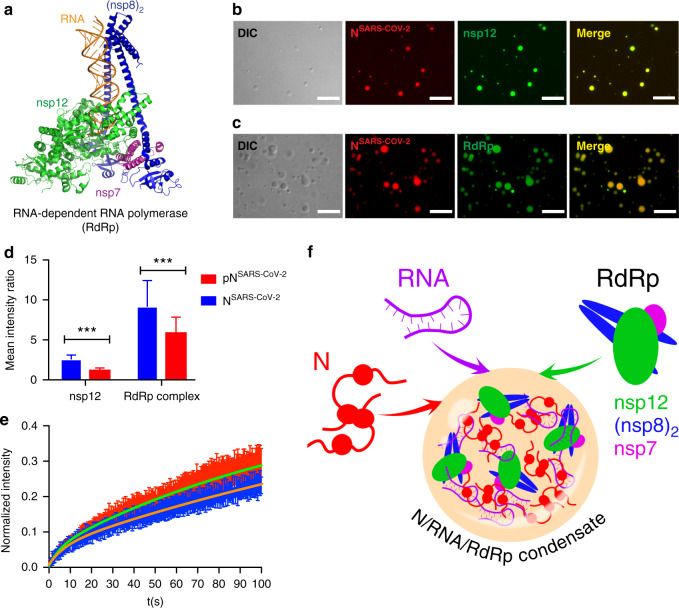
The RNA-dependent RNA polymerase complex of SARS-CoV-2 concentrates in RNA/nucleocapsid protein-droplets. **a** Structure of the RNA-bound RdRp-complex formed by the SARS-CoV-2 non-structural proteins nsp12 (green), nsp7 (magenta) and nsp8 (blue) in 1:1:2 stoichiometry (PDB code: 6YYT)^29^. **b** Fluorescence and DIC microscopy show the recruitment of Alexa Fluor 488 labeled nsp12 (green), the catalytic subunit of the RdRp-complex, into N^SARS-CoV-2^/polyU droplets (N^SARS-CoV-2^ in red). **c** Active SARS-CoV-2 RdRp-complex bound to a fluorescein-labeled minimal RNA hairpin concentrates inside of N^SARS-CoV-2^/polyU droplets. In (**b**) and (**c**) droplets of 50 µM N^SARS-CoV-2^ and 1 µM polyU were prepared in 20 mM NaPi, pH 7.5, and visualized by the addition of a small amount of Alexa Fluor 594 labeled N^SARS-CoV-2^ protein. Scale bars are 10 µm in (**b**) and (**c**). Micrographs are representative of three independent biological replicates. **d** Recruitment of nsp12 and RdRp complex into polyU-induced droplets of unmodified (blue) and SRPK1-phosphorylated (red) N^SARS-CoV-2^. Mean values and standard deviation are shown (*n* = 100 droplets). Two-sided t-test with *P* value set to < 0.05 for statistical significance, ***<0.001, **<0.002 and *<0.033, ns < 0.12. **e** FRAP of nsp12 after recruitment into N^SARS-CoV-2^/polyU (blue) and SRPK1-phosphorylated N^SARS-CoV-2^/polyU (red) droplets. Error bars represent the standard deviation for averaged 10 and 11 curves for unmodified and phosphorylated N^SARS-CoV-2^, respectively. (**f**) Schematic representation of the LLPS-based formation of N/RNA/RdRp-condensates as protein/RNA-dense sites for viral transcription.

LLPS provides a cooperative mechanism to locally increase protein and RNA concentrations^11,12^. In addition, protein/RNA condensates can recruit additional proteins to promote reactions. To investigate if the RNA-dependent RNA polymerase (RdRp; Fig. 5a) concentrates within N^SARS-CoV-2^/RNA droplets, we recombinantly prepared the non-structural protein (nsp) 12 of SARS-CoV-2, together with the accessory sub-units nsp7 and nsp8, which are required for transcription^29^. First, we used nsp12, in order to investigate if the catalytic component of RdRp is recruited to N^SARS-CoV-2^/polyU droplets. Fluorescence microscopy revealed strong nsp12 fluorescence inside the droplets (Fig. 5b). Next, nsp12, nsp7, and nsp8 were reconstituted in a 1:1:2 stoichiometry together with a RNA template-product duplex, which carried fluorescein at the 5’ end^29^. The RdRp/RNA-complex was added to preformed N^SARS-CoV-2^/polyU droplets into which the RdRp/RNA-complex was recruited (Fig. 5c). High local concentrations of RdRp and N^SARS-CoV-2^ were reached (Supplementary Fig. 9).

We then investigated the influence of phosphorylation of N^SARS-CoV-2^ by the kinase SRPK1 on the recruitment of nsp12 and the RdRp/RNA-complex into N^SARS-CoV-2^/RNA droplets. The analysis showed that both nsp12 and the RdRp/RNA-complex partition less into the droplets formed by SRPK1-phosphorylated N^SARS-CoV-2^ (Fig. 5d). This indicates that N^SARS-CoV-2^ not only interacts with RNA but also directly binds to nsp12 and the nsp12/N^SARS-CoV-2^-interaction is attenuated by phosphorylation of the SR-region of N^SARS-CoV-2^. This mechanism was further supported by a more rapid recovery of fluorescence after photobleaching of nsp12 in droplets formed by SRPK1-phosporylated N^SARS-CoV-2^ (Fig. 5e). The data suggest that RNA-driven condensation of N^SARS-CoV-2^ provides a mechanism for bringing together components of the viral replication machinery (Fig. 5f). In agreement with this proposed mechanism, N protein of SARS-CoV colocalizes intracellularly with replicase components^30^.

## Discussion

Our study shows that the nucleocapsid protein of the SARS-CoV-2 virus undergoes RNA-induced liquid-liquid phase separation. Although nucleocapsid assembly can occur outside of liquid-like compartments, it was shown that the rate of assembly is increased when the nucleocapsid protein of Measles virus is concentrated through LLPS^15^. In addition, N^SARS-CoV-2^ interacts with human ribonucleoproteins^21^, which are found in several LLPS-driven cytosolic protein/RNA granules, suggesting that N^SARS-CoV-2^ might modulate protein/RNA granule formation in order to maximize viral replication^31^. In agreement with such activity, we showed that N^SARS-CoV-2^ translocates to stress granules in stressed cells.

We demonstrate that N^SARS-CoV-2^ LLPS promotes cooperative association of the RNA-dependent RNA polymerase complex with polyU RNA in vitro. This suggests that SARS-CoV-2 uses LLPS-based mechanisms similar to transcription hubs in cellular nuclei^32,33^ to enable high initiation and elongation rates during viral transcription. Because the replication machinery of coronaviruses is membrane-associated^34^, it will furthermore be interesting to investigate if the SARS-CoV-2 glycoprotein M, which binds nucleocapsid protein^4^, causes tethering of N^SARS-CoV-2^/RdRp/RNA-condensates to host cell membranes.

Taken together the data suggest that inhibition of the RNA-induced phase separation of the nucleocapsid protein of SARS-CoV-2 provides a viable and novel strategy for the design of therapeutics to treat Covid-19.

## Methods

### Materials

Alexa Fluor 594 conjugated anti G3BP1 antibody was purchased from Santa Cruz Biotechnology (sc-365338 AF594). Digitonin was from Merck (CAS 11024-24-1). PolyU potassium salt (800 kDa) was purchased from Sigma-Aldrich (CAS Number 27416-86-0). Recombinant nucleocapsid protein of SARS-CoV-2 (Catalog No: Z03488-1) and SRPK1 kinase (Catalog No: PV4215) were purchased from GenScript and ThermoFisher, respectively. The SR peptide, comprising residues 182-197 of the nucleocapsid protein of SARS-CoV-2, was produced by Fmoc-solid-phase synthesis using an automated microwave peptide synthesizer (Liberty 1, CEM) with acetyl‐ and amide protection groups at the N‐ and C‐termini, respectively, and further purified by reverse-phase high-performance liquid chromatography (LC). Peptide masses were determined by LC-MS using an Acquity Arc System (WATERS) with SQD2-mass-detector.

### Purification of nsp12, nsp7, nsp8 and formation of RdRp complex

Nsp12, nsp7, and nsp8 were expressed and purified as previously described^29^: nsp12 was expressed in Hi5 insect cells using the pFastBac-His-MBP-SCoV2-nsp12 plasmid (Addgene #154759). Cells were collected by centrifugation, resuspended in lysis buffer (300 mM NaCl, 50 mM Na-HEPES pH 7.4, 10% (v/v) glycerol, 30 mM imidazole pH 8.0, 5 mM β-mercaptoethanol, 0.284 μg ml^−1^ leupeptin, 1.37 μg ml^−1^ pepstatin, 0.17 mg ml^−1^ PMSF and 0.33 mg ml^−1^ benzamidine, 3 mM MgCl_2_) and immediately sonicated. Lysates were cleared by centrifugation, filtered (0.8 μm), and applied to a HisTrap HP 5 ml (GE Healthcare) preequilibrated in lysis buffer. The protein was eluted from the HisTrap column directly onto an XK column 16/20 (GE Healthcare), prepacked with amylose resin (New England Biolabs), using nickel elution buffer (300 mM NaCl, 50 mM Na-HEPES pH 7.4, 10% (v/v) glycerol, 500 mM imidazole pH 8.0, 3 mM MgCl_2_ and 5 mM β-mercaptoethanol). Protein was eluted from the amylose column using amylose elution buffer (300 mM NaCl, 50 mM Na-HEPES pH 7.4, 10% (v/v) glycerol, 116.9 mM maltose, 30 mM imidazole pH 8.0 and 5 mM β-mercaptoethanol). Peak fractions containing nsp12 were pooled, cleaved with His-tagged TEV (12 h), and applied to a HisTrap column to remove uncleaved nsp12, 6×His–MBP, and TEV. The flow-through containing nsp12 was applied to a HiTrap Heparin 5 ml column (GE Healthcare). The flow-through containing nsp12 was collected, concentrated, and applied to a HiLoad S200 16/600 pg equilibrated in the size-exclusion buffer (300 mM NaCl, 20 mM Na-HEPES pH 7.4, 10% (v/v) glycerol, 1 mM MgCl2, 1 mM TCEP). Peak fractions were pooled, concentrated until 102 μM, aliquoted, flash-frozen, and stored at −80 °C until use.

Nsp7 and nsp8 were expressed in E.coli using the pET-His-SCoV2-nsp7 plasmid (Addgene #154757) and pET-His-CoV2-nsp8 plasmid (Addgene #154758), respectively. Proteins were overexpressed in E. coli BL21 (DE3) RIL cells, grown in LB medium, collected by centrifugation, resuspended in lysis buffer (300 mM NaCl, 50 mM Na-HEPES pH 7.4, 10% (v/v) glycerol, 30 mM imidazole pH 8.0, 5 mM β-mercaptoethanol, 0.284 μg ml−1 leupeptin, 1.37 μg ml−1 pepstatin, 0.17 mg ml−1 PMSF and 0.33 mg ml−1benzamidine) and immediately sonicated. Both nsp8 and nsp7 were purified separately using the same purification procedure. Lysates were cleared by centrifugation and the supernatants applied to a HisTrap HP 5 ml column (GE Healthcare) preequilibrated in lysis buffer. The sample was eluted using nickel elution buffer (150 mM NaCl, 50 mM Na-HEPES pH 7.4, 10% (v/v) glycerol, 500 mM imidazole pH 8.0, and 5 mM β-mercaptoethanol) and the eluted protein dialyzed in dialysis buffer (150 mM NaCl, 50 mM Na-HEPES pH 7.4, 10% (v/v) glycerol and 5 mM β-mercaptoethanol) in the presence of His-tagged TEV (12 h at 4 °C). After adding imidazole pH 8.0, the sample was applied to a HisTrap HP 5 ml column (GE Healthcare). The flow-through with the protein of interest was then applied to a HiTrap Q 5 ml column (GE Healthcare) and the concentrated new flow-through applied to a HiLoad S200 16/600 pg equilibrated in size exclusion buffer (150 mM NaCl, 20 mM Na-HEPES pH 7.4, 5% (v/v) glycerol, 1 mM TCEP). Peak fractions were pooled, concentrated, aliquoted, flash-frozen, and stored at −80 °C until use. Protein purifications were performed at 4 °C.

For the preparation of the fluorescently labeled RNA, an RNA scaffold for RdRp/RNA complex formation was annealed by mixing equimolar amounts of two RNA strands (5’- rUrUrUrUrCrArUrGrCrArUrCrGrCrGrUrArG rGrCrUrCrArUrArCrCrGrUrArUrUrGrArGrA -3’; 56-FAM/rUrCrUrCrArArUrArCrGrGrUrArUrGrArGrC CrUrArCrGrCrG-3’) (IDT Technologies) in annealing buffer (10 mM Na-HEPES pH 7.4, 50 mM NaCl) and heating to 75 °C, followed by step-wise cooling to 4 °C. For complex formation, 2.45 nmol of purified nsp12 was mixed with a 1.1-fold molar excess of RNA scaffold and 3-fold molar excess of each nsp8 and nsp7. After incubation at room temperature for 10 min, the complex was subjected to size exclusion chromatography on a Superdex 200 Increase 3.2/300 equilibrated with complex buffer (20 mM Na-HEPES pH 7.4, 100 mM NaCl, 1 mM MgCl_2_, 1 mM TCEP). Peak fractions with a volume of approx. 125 µL (absorbance at 280 nm of 2.7 AU, 10 mm path length) corresponding to a nucleic acid-rich high-molecular-weight population (as judged by absorbance at 260 nm) were pooled and used for subsequent experiments.

### Turbidity measurements

Phase diagrams of non-phosphorylated and phosphorylated N^SARS-CoV-2^ at different concentrations were determined using a NanoDrop spectrophotometer (ThermoFisher Scientific, Invitrogen). Increasing concentrations of polyU (0–2 µM) were added immediately before the experiments, followed by thoroughly pipetting and measurement of turbidity at 350 nm UV–Vis. Averages turbidity values were derived from measurements of three independent, freshly prepared samples.

### Microscopy

For fluorescence microscopy, proteins were labeled using Alexa-fluor 488^TM^ (green) or Alexa-fluor 594^TM^ (red) microscale protein labeling kits (ThermoFisher Scientific, Invitrogen). Small amounts (~0.3 µl) of fluorescently-labeled N^SARS-COV-2^ were premixed with unlabeled N^SARS-COV-2^ and diluted to 50 µM final concentration with NaPi 20 mM buffer, pH 7.5. PolyU was added to the mixture to reach a final concentration of 1 µM. A total of 5 µl of the sample was subsequently loaded onto a slide and covered with a 18 mm coverslip. DIC and fluorescent micrographs were acquired on a Leica DM6B microscope with a 63x objective (water immersion) and processed using Fiji software (NIH). For RNA recruitment assays, fluorescently labeled RNA was premixed with 1 µM polyU and subsequently added to the mixture of fluorescently labeled/unlabeled N^SARS-COV-2^. For nsp12/RdRp/RNA recruitment assays, the fluorescently labeled component (FAM-labeled RNA scaffold, nsp12, or RdRp/RNA-complex) was premixed with 1 µM polyU, followed by addition to a mixture of fluorescently labeled/unlabeled N^SARS-COV-2^.

### Quantification of the recruitment of fluorescently labeled RNA, nsp12, and RdRp complex into unmodified and SRPK1-phosphorylated N^SARS-COV-2^

The partition coefficient of either fluorescently labeled RNA, nsp12 or the RdRp complex was calculated from microscopy data using the FIJI software. For each of the above-mentioned conditions, the partition coefficient was calculated as:,

1 $$Partition\,coefficient = \frac{{Fluorescence\,Mean\,intensity}}{{Background\,Mean\,intensity}}$$

where the fluorescence mean intensity is the averaged droplet intensity calculated by defining its area and the background mean intensity corresponds to the averaged intensity of the background. For quantification of the partition coefficient of fluorescently labeled RNA, nsp12, or the RdRp complex, two independent samples were used and a total amount of 100 droplets per condition were analyzed. A two-tailed t-test was used to compare the partition coefficient values obtained for either fluorescently labeled RNA, nsp12, or the RdRp complex recruited into N^SARS-CoV-2^ (used as a control group) and phosphorylated N^SARS-CoV-2^. A *P* value < 0.05 was set for statistical significance. The t-test was performed in Graph Prism.

### Fluorescence recovery after photobleaching (FRAP)

Dynamics of N^SARS-CoV-2^ molecules in the phase-separated state were investigated by FRAP analysis. As described above, N^SARS-CoV-2^ phase separation was induced using 50 µM N^SARS-CoV-2^ and 1 µM polyU in 20 mM sodium phosphate buffer (NaPi), pH 7.5. For comparison of the kinetics of unmodified and SRPK1 phosphorylated N^SARS-CoV-2^ protein, droplets of 30 µM nucleocapsid protein induced by the addition of 0.4 µM polyU were used. Droplet movement was reduced through the addition of 5 % dextran T500 (Pharmacosmos). The sample was spiked with a small amount of Alexa Fluor 488 lysine labeled unmodified or SRPK1 phosphorylated N^SARS-CoV-2^, or labeled nsp12 in the case of investigating the kinetics of nsp12 in droplets of unmodified or SRPK1 phosphorylated N^SARS-CoV-2^ (30 µM nucleocapsid protein, 0.3 µM polyU in 20 mM sodium phosphate buffer (NaPi), pH 7.5.). Labeled proteins were photobleached and followed during recovery. FRAP recordings were done on freshly formed droplets settled down on the glass slide (early time points; time of recording <15 min) and after one hour of sample incubation on the glass (late time points). For FRAPs of unmodified and SRPK1 phosphorylated N^SARS-CoV-2^ recordings were done in late time points.

FRAP experiments were recorded on a Leica TCS SP8 confocal microscope using a 63x objective (oil immersion) and a 488 argon laser line. A circular region of ~4 µm in diameter was chosen in a region of homogenous fluorescence away from the droplet boundary and bleached with five iterations of full laser power. Recovery was imaged at low laser intensity (5%). 100 frames were recorded with one frame per 523 ms. Pictures were analyzed in FIJI software (NIH) and FRAP recovery curves were calculated using standard methods on the basis of fluorescence intensities measured for prebleaching, bleached, and reference ROI. The prebleaching ROI was a selected region in the droplet before bleaching; the bleached ROI corresponded to the bleached area while the reference ROI corresponded to an area that did not experience bleaching. The fluorescence intensity measured for each of the described ROIs was corrected by background substraction: a region where no fluorescence was detected was used to calculate the background. Thus, the FRAP recovery was calculated as:

2 $$FRAP = \frac{{I_{Bleached} - I_{background}}}{{I_{Av.Prebleaching}}}$$

The value obtained was then corrected by multiplication with the acquisition bleaching correction factor (ABCF), which was calculated according to:

3 $$ABCF = \frac{{I_{reference} - I_{background}}}{{I_{Av.Reference}}}$$

Finally, the curves were normalized according to:

4 $$Normalization = \frac{{I_{\left( t \right)} - min.Intensity\,Value}}{{1 - min.Intensity\,Value}}$$

Values were averaged from six recordings for both early and late time points and the resulting FRAP curves ± standard deviation (std) were fitted for the early time points to a mono-exponential function:

5 $$y = a \ast (1 - exp( - b \ast x)) + c$$

For the late time points, a bi-exponential function provided the best fitting:

6 $$y = a \ast \left( {1 - exp\left( { - b \ast x} \right)} \right) + y + a2 \ast (1 - exp( - b2 \ast x)) + c$$

The mobile and immobile fractions were calculated using the parameters a and c derived from each fitting, according to the following equations:

7 $$Mobile\,fraction = a + c$$

8 $$Immobile\,fraction = 1 - a - c$$

### Stress granule co-localization and FRAP

HeLa cells (DSMZ-German Collection of Microorganisms and Cell Cultures GmbH, ACC 57) were grown in an incubator at 37 °C in a humidified atmosphere with 5% CO_2_. One day before the stress granule co-localization assay and FRAP measurements cells were seeded in 96-well CELLview^TM^ slides (Greiner Bio-One) with glass bottom suitable for imaging so that on the day of the measurement cells reached ~50% of confluence. Stress granule formation was induced by treatment with 0.5 mM sodium arsenite for 60 minutes. Subsequently, cell membranes were permeabilized by incubation in cell permeabilization buffer (20 mM HEPES-KOH pH 7.5, 120 mM KOAc, 5 mM Mg(OAc)_2_, 250 mM sucrose) supplemented with 60 µg/ml digitonin for 40 s. After washing cells four times with 100 µL of cell permeabilization buffer live recording was started on a Leica TCS SP8 confocal microscope using a 63x objective (oil immersion) and 488 argon laser or 561 laser lines. A mixture of 0.5 µm Alexa Fluor 488 lysine labeled N^SARS-CoV-2^ and 1:100 Alexa Fluor 594 conjugated G3BP1 antibody (Abcam, ab217225) in cell permeabilization buffer was added to the cells and movies were recorded with 512 ×512 pixel resolution at 1000 Hz speed and 1 s per frame for about 2–3 min. For FRAP measurements, individual stress granules were marked and photobleached with five iterations of full 488 argon laser power. Recovery was imaged at low laser intensity (8%). 200 frames were recorded with one frame per 523 ms. Pictures were analyzed in FIJI software (NIH) and FRAP recovery curves ± standard deviation were calculated and fitted using bi-exponential fit as described above. Non-bleached stress granules were used as a reference for calculations.

### In vitro phosphorylation

Phosphorylation of a stock of 850 µM SR-peptide (1766.8 Da) was performed by incubation with 0.15 µM SRPK1 kinase at 23 °C overnight in a buffer containing 4 mM ATP, 5 mM MgCl_2_, 1 mM DTT, and 5 mM EGTA. Because of the intrinsically disordered nature of the peptide, inactivation of the kinase was achieved by incubation of the sample at 65 °C for 20 min, followed by centrifugation at 15,000 × *g* for 30 min. Residual ATP, MgCl_2,_ and EGTA were removed by HPLC followed by mass spectrometry. Phosphorylation of 100 µM unlabeled N^SARS-COV-2^ was performed by incubation with 0.5 µM of SRPK1 kinase. The reaction mixture was incubated at 23 °C overnight in a buffer containing 8 mM ATP, 5 mM MgCl_2_, 1 mM DTT, and 5 mM of EGTA. Residual ATP, MgCl_2_, and EGTA were removed by 4 times buffer exchange using a Vivaspin 500.5 molecular weight cut-off (Sartorius, Göttingen). Samples were loaded onto a SDS-PAGE gel to confirm phosphorylation.

### Mutation frequency analysis

To examine the mutation frequency in SARS-CoV-2 sequences we used the database and resources from the China National Center for Bioinformation, 2019 Novel Coronavirus Resource (https://bigd.big.ac.cn/ncov?lang=en; downloaded June 7, 2020, with 42,176 genome sequences). The mutations between the genome positions 28274 and 29530 were analyzed to get the mutation frequency for each codon.

### NMR spectroscopy

One-dimensional (1D) ^1^H NMR experiments and two-dimensional (2D) ^1^H-^1^H TOCSY, NOESY, and ^1^H-^15^N/^1^H-^13^C heteronuclear single quantum coherence (HSQC) experiments of the SR-peptide (residues A182-S197) of N^SARS-CoV-2^ were acquired at 5 °C on a Bruker 700 MHz spectrometer equipped with a triple-resonance 5 mm cryogenic probe using the software Top Spin 3.5 (Bruker). The peptide concentration was 4 mM for resonance assignment and 200 µM for the interaction analysis with polyU (800 kDa). Samples were in 50 mM NaP, 0.01% NaN_3_ and 5% D_2_O. Spectra were processed with TopSpin 3.6 (Bruker) and analyzed using Sparky^35^. Secondary structure was analyzed subjecting experimental HA, HN, N, CA and CB chemical shifts to TALOS+^36^. The chemical shift perturbation (CSP) for the peptide residues is the one of the NH protons from the TOCSY experiment. The CSP error is based on the resolution of the spectra.

### Molecular dynamics simulations

Starting structures of the SR-peptides were built in the PyMOL Molecular Graphics System (Version 1.8.4.0, Schrödinger, LLC), those of the RNA molecules using the RNA modeling software SimRNA^37^. Initially, the different mixtures were equilibrated with 50,000 steps of energy minimization. To further equilibrate the system, 100 ps each of volume (NVT) and pressure (NPT) equilibration were performed without position restrains in order to have different starting points in each simulation. The MD simulations were carried out in GROMACS (version 2018.3) using the AMBER99SB-ILDN force field and the TIP3P water model at a temperature of 300 K, 1 bar of pressure and with a coupling time (ζT) of 0.1 ps. The mixtures were solvated in water with 150 mM NaCl, ensuring overall charge neutrality. The particle mesh Ewald algorithm was used for calculation of the electrostatic term, with a radius of 16 Å for the grid-spacing and Fast Fourier Transform. The cut-off algorithm was applied for the non-coulombic potential with a radius of 10 Å. The LINCS algorithm was used to contain bonds and angles. MD simulations were performed during 1 or 100 ns in 2 fs steps and saving the coordinates of the system every 10 ps. The force field parameters for the phosphorylated amino acids were taken from^38^. The number of contacts and secondary structure over the simulation trajectory were analyzed using the PyMOL Molecular Graphics System (Version 1.8.4.0, Schrödinger, LLC). To get error bars, 5 repetitions were done for each 1 ns simulation. For 100 ns simulations the error is the standard deviation over the trajectory.

### Reporting summary

Further information on experimental design is available in the Nature Research Reporting Summary linked to this paper.

## Supplementary information

Supplementary Information

Description of Additional Supplementary Files

Supplementary Movie 1

Reporting summary

## Data Availability

NMR assignments are available in the BMRB (code 50379). The mutation frequency from SARS-CoV-2 sequences were obtained from the database and resources of the China National Center for Bioinformation, 2019 Novel Coronavirus Resource (https://bigd.big.ac.cn/ncov?lang=en; downloaded June 7, 2020, with 42,176 genome sequences). Authors can confirm that the rest of the relevant data are included in the paper and/or its supplementary information files. Other data that support the findings of this study are available from the corresponding author upon reasonable request. Source data are provided with this paper.

## Source data

Source Data
