# Supplementary material for: Nucleocapsid protein of SARS-CoV-2 phase separates into RNA-rich polymerase-containing condensates
Source: Nat Commun. 2020 Nov 27;11:6041. doi: 10.1038/s41467-020-19843-1 (PMC7699647; doi:10.1038/s41467-020-19843-1)
Supplement: Supplementary file 1 — Supplementary Information [file 41467_2020_19843_MOESM1_ESM.pdf]

Supplementary Information for

Nucleocapsid protein of SARS-CoV-2 phase separates into RNA-rich polymerase-containing condensates

Adriana Savastano, Alain Ibáñez de Opakua, Marija Rankovic, and Markus Zweckstetter

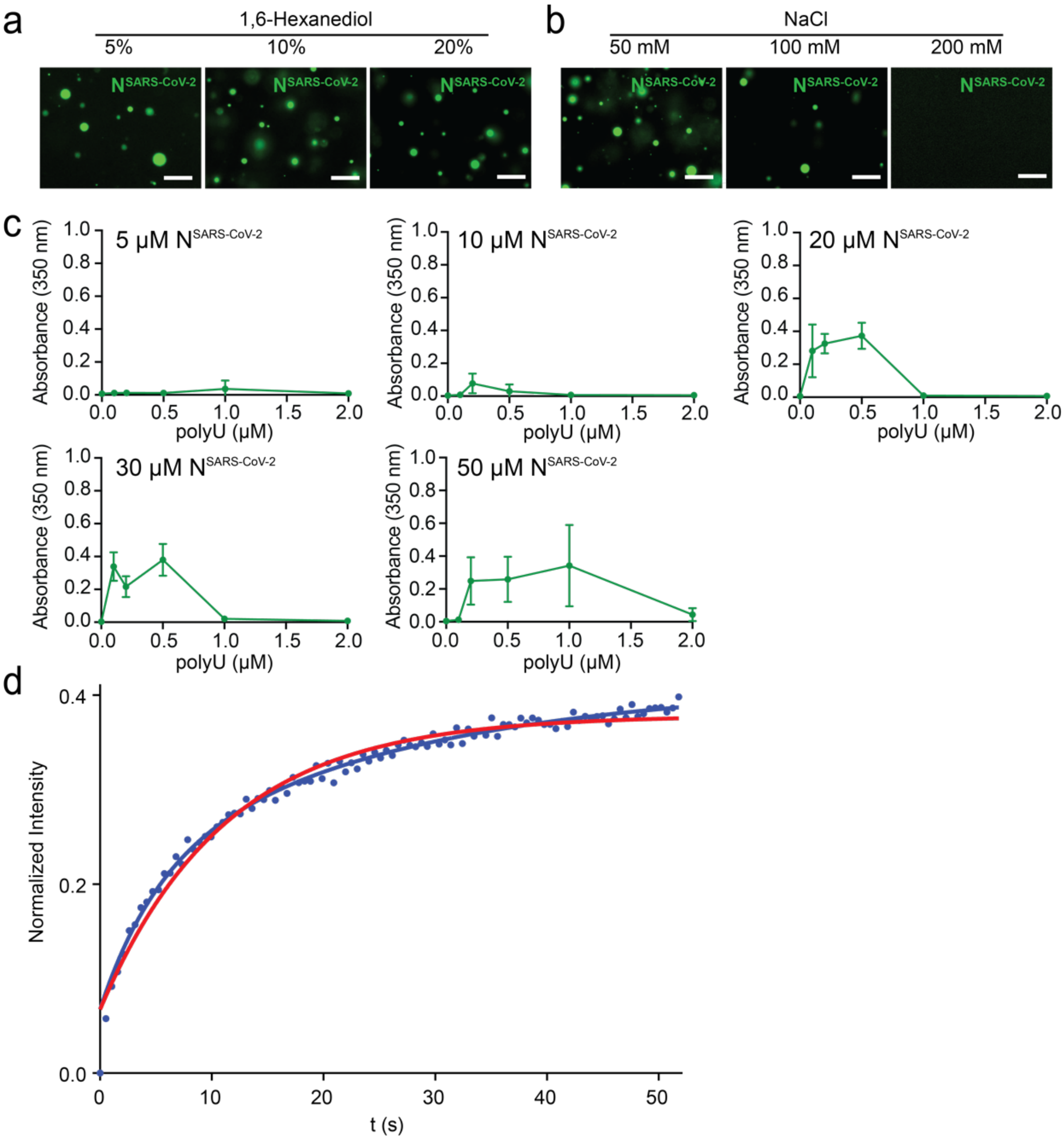

Supplementary Fig. 1. RNA-induced LLPS of N$^{SARS\text{-}CoV\text{-}2}$. **a-b,** Fluorescence microscopy of droplets formed by 50 µM N$^{SARS\text{-}CoV\text{-}2}$ and 1 µM polyU in 20 mM NaPi, pH 7.5, at increasing concentrations of 1,6-hexanediol (a) or NaCl (b). Scale bars, 20 µm. Micrographs are representative of two independent biological replicates. **c,** Turbidity at 350 nm of solutions of N$^{SARS\text{-}CoV\text{-}2}$ in 20 mM NaPi, pH 7.5, at different protein concentrations (5-50 µM) and increasing concentrations of polyU. Average values from three independent measurements are shown and also displayed in Figure 1b. Error bars, std. **d,** Fit of a mono-exponential (red) and bi-exponential (blue) function to FRAP data obtained for N$^{SARS\text{-}CoV\text{-}2}$/polyU droplets incubated for one hour.

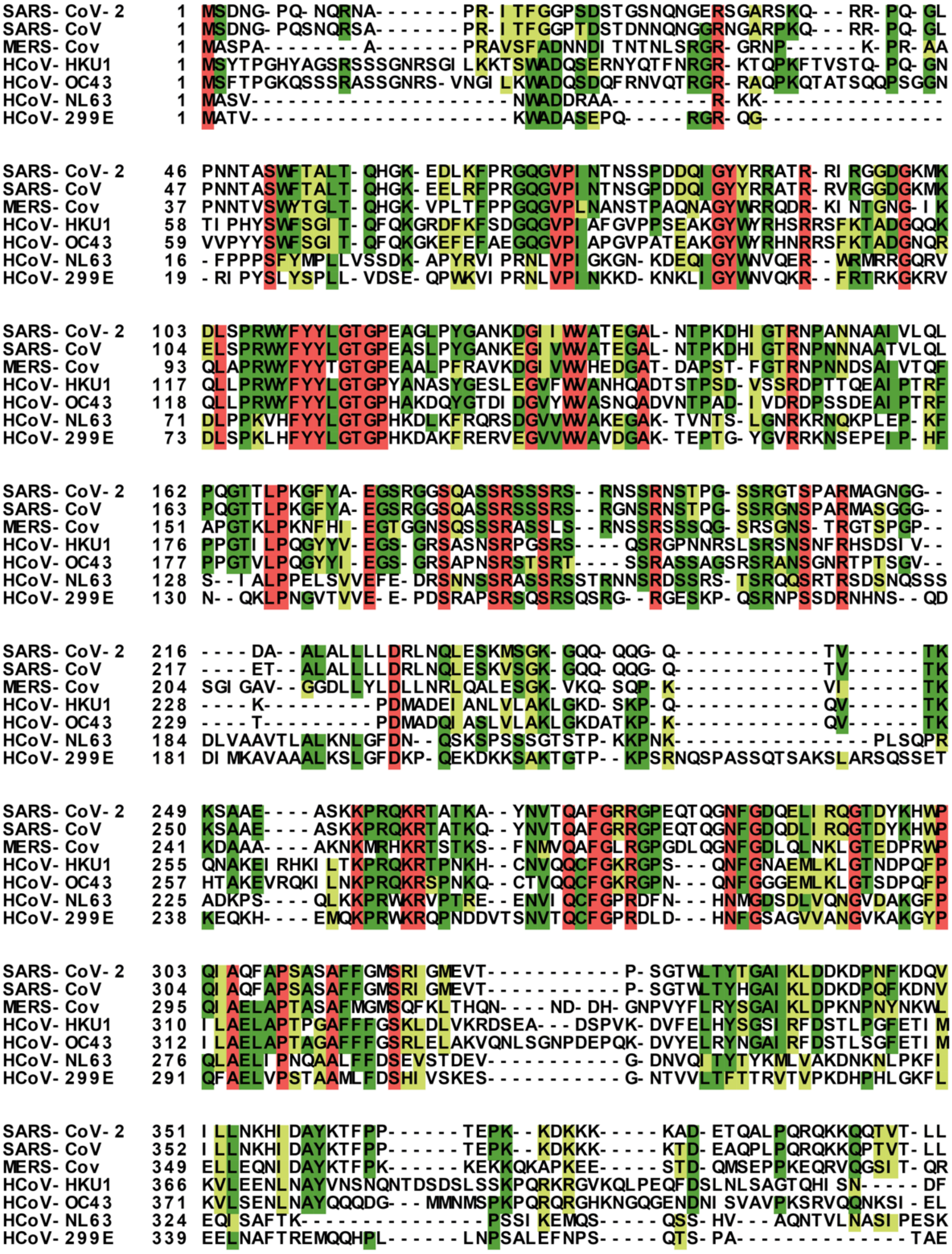

Supplementary Fig. 2. Sequence alignment of the seven human coronaviruses. The following sequences were used: SARS-CoV-2, *YP_009724397.2*; SARS-CoV, *NC_004718*; MERS-CoV, *NC_019843*; HCoV-HKU1, *NC_006577*; HCoV-OC43, *KF530099.1*; HCoV-299E, *NC_002645*; HCoV-NL63, *NC_005831*. Identical residues are marked in red, homologues residues in dark and light green.

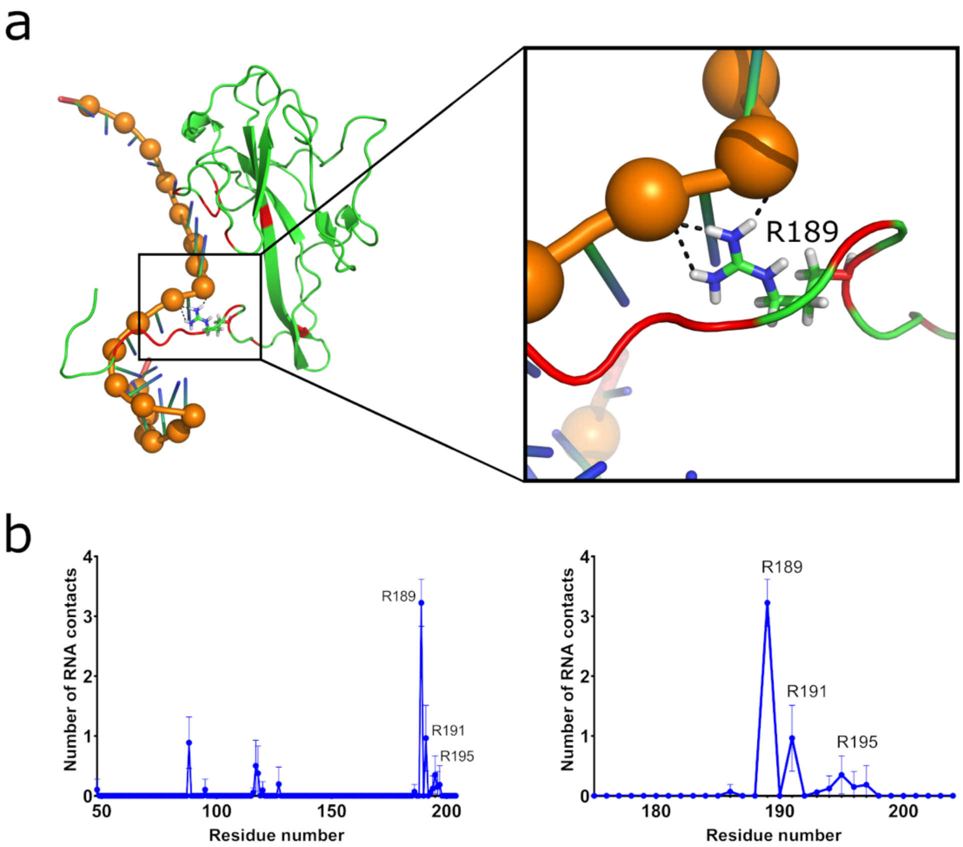

Supplementary Fig. 3. Molecular dynamics simulations of RBD-SR (residues 48-204 of N$^{SARS\text{-}CoV\text{-}2}$) with RNA. a, Final structure at the end of one of the simulations (1 ns) highlighting the contact of R189 with polyU (zoom on the right). The orange spheres represent phosphate groups and the red color in the protein structure highlights residues, for which polar contacts with RNA were most frequent during the simulation. **b,** Averaged number of RNA contacts from three independent 1 ns simulations in the whole sequence (left) and in the SR-rich region (right). Error bars represent standard deviation.

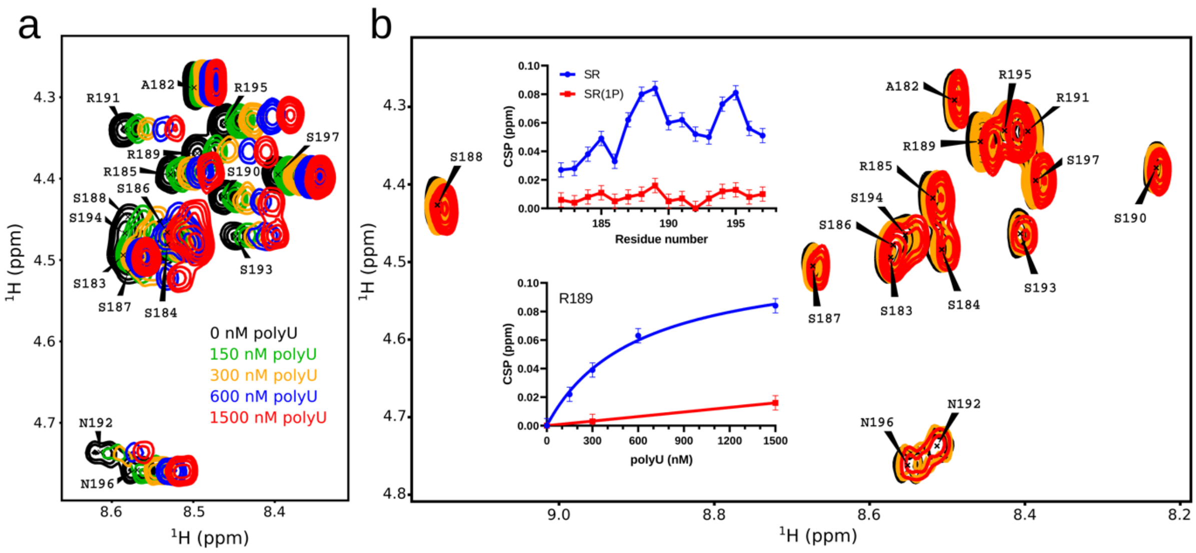

Supplementary Fig. 4. NMR spectroscopy of the interaction of polyU with the SR-peptide comprising residues 182-197 of N$^{SARS\text{-}CoV\text{-}2}$. **a,** Selected regions from 2D TOCSY experiments of the SR-peptide at five different concentrations of polyU (0, 150, 300, 600 and 1500 nM). Resonance assignments are indicated. **b,** TOCSY spectra of the SPRK1 single-phosphorylated peptide at three different concentrations of polyU (0, 300 and 1500 nM). The positive charges of the SR-peptide are compensated by the negative charges of polyU at around 300 nM polyU. Spectral color code as in (a). The residue-specific chemical shift perturbation (CSP) observed for each peptide at 1500 nM of polyU are shown in the top plot. The change in R189 CSP with increasing polyU concentration is shown in the bottom plot. Data for the non-phosphorylated SR-peptide are shown in blue, for the SPRK1 single-phosphorylated peptide in red. The CSP error is based on the resolution of the spectra.

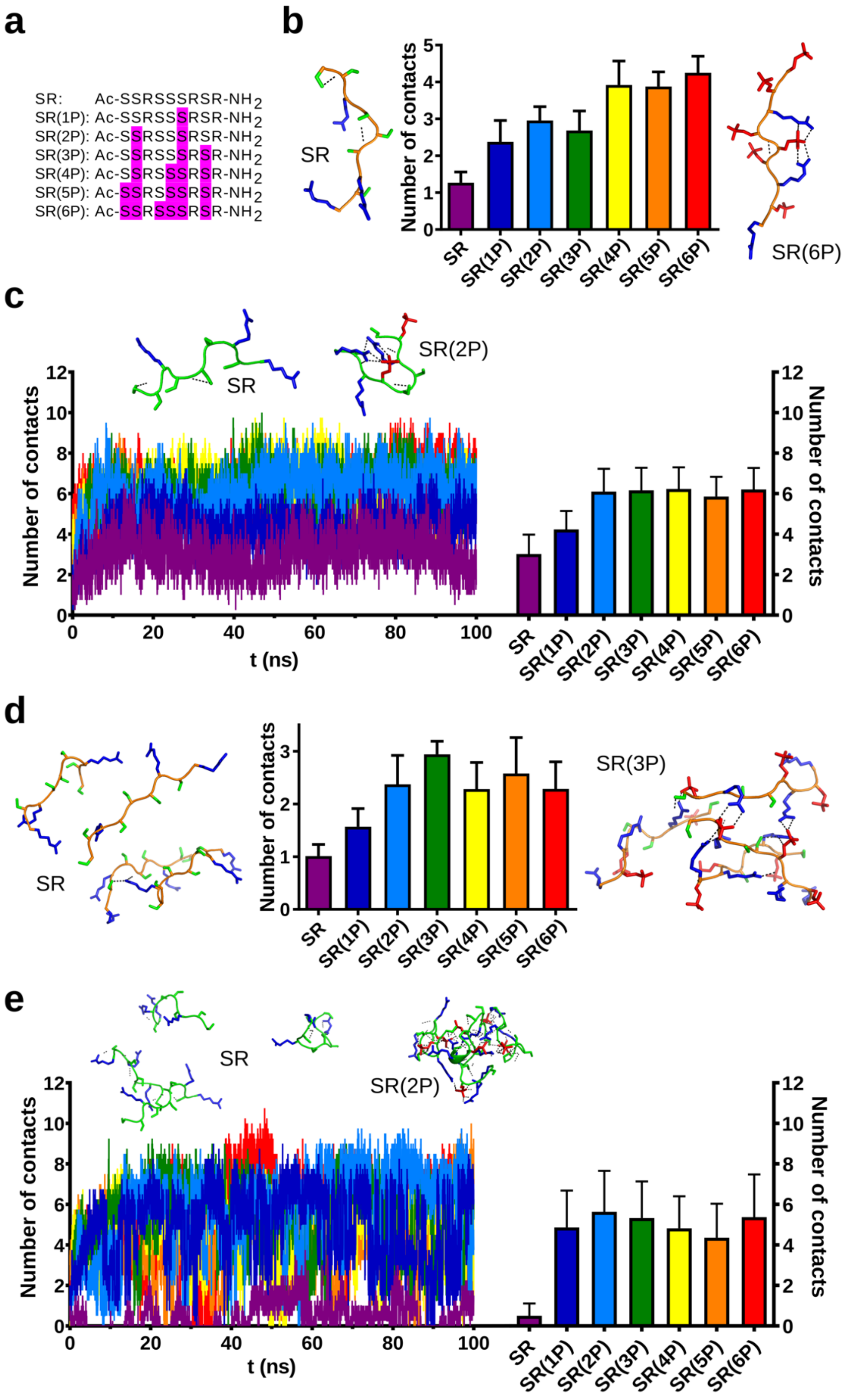

Supplementary Fig. 5. Intra- and intermolecular interactions observed in MD simulations of SR-peptides comprising residues 183-191 of N$^{\text{SARS-CoV-2}}$. **a,** Amino acid sequences of the SR-peptides with phosphorylated serine residues highlighted in magenta. **b,** Number of

intramolecular polar contacts per peptide for each SR-peptide displayed as the average and standard deviation of five independent 1 ns simulations. Snapshots of the non-phosphorylated (left) and fully-phosphorylated SR-peptide (right) are shown. **c,** Number of intramolecular polar contacts over the trajectory of five 100 ns simulations for the same set of peptides and the averages with standard deviations are represented at the right. MD snapshots of the non-phosphorylated (left) and 2-times phosphorylated (right) peptide are shown above. **d**, Number of intermolecular polar contacts per peptide for each SR-peptide displayed as the average and standard deviation of five independent 1 ns simulations. Snapshots of non-phosphorylated (left) and 3-times phosphorylated SR-peptides (right). **e**, Number of intermolecular polar contacts over the trajectory of 100 ns simulations and the averages with standard deviations are represented to the right. MD snapshots of the non-phosphorylated (left) and 2-times phosphorylated (right) peptide are shown above. MD simulations were performed with four identical peptides in the water box. Salt bridges between the phosphate groups (red) and the arginine side-chains (blue) are marked by dashed lines.

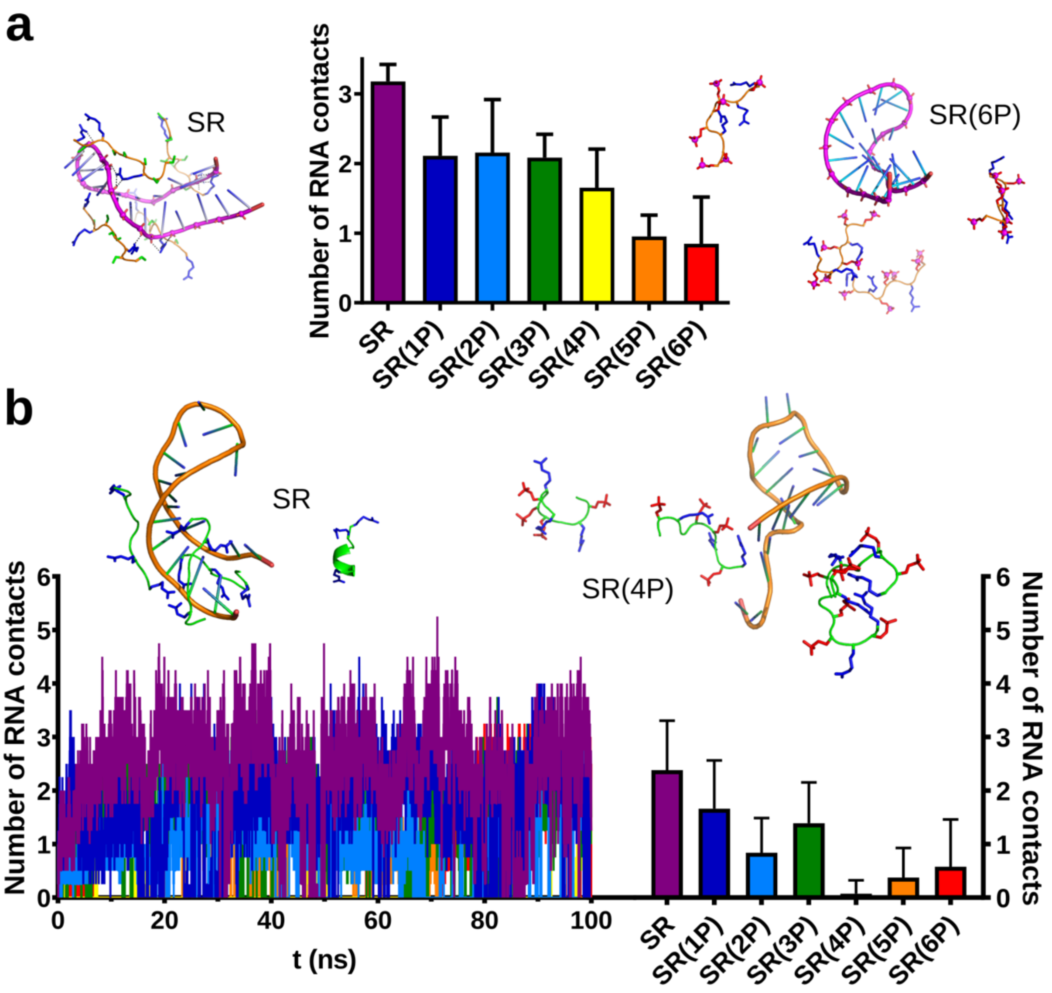

Supplementary Fig. 6. RNA-interactions observed in MD simulations of SR-peptides comprising residues 183-191 of N$^{SARS-CoV-2}$. a, The number of polar contacts of the different SR-peptides (see Supplementary Fig. 4a) with a structured RNA derived from the viral genome of SARS-CoV-2 are displayed as the average and standard deviation of five independent 1 ns simulations. MD snapshots of the RNA-interaction of the non-phosphorylated (left) and fully-phosphorylated (right) peptide are shown at both sides. **b**, Number of polar contacts with RNA over the trajectory of five 100 ns simulations for the same set of SR-peptides. Averages with standard deviations are represented at the right. MD snapshots of the non-phosphorylated (left) and 4-times phosphorylated (right) peptide are shown above. Arginine side chains in blue.

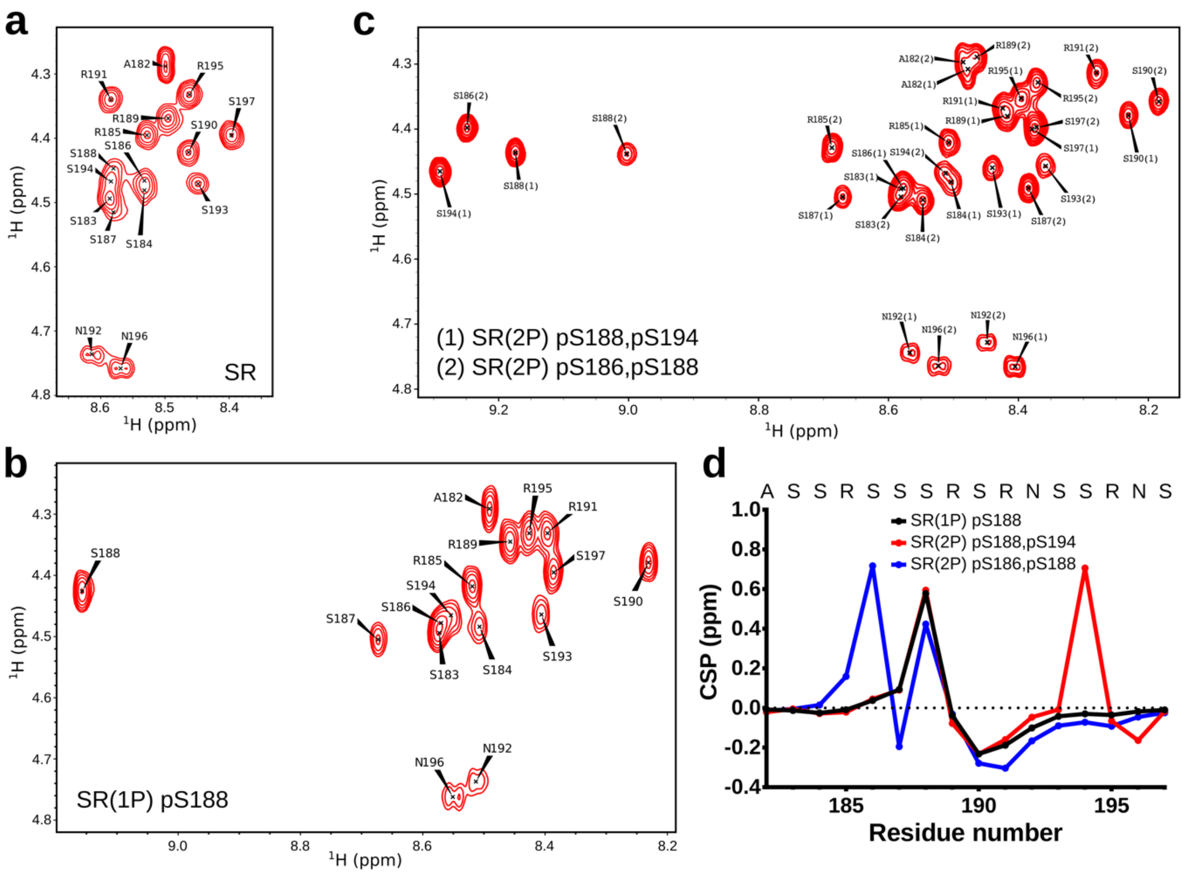

Supplementary Fig. 7. Assignment of TOCSY spectra of SR-peptide comprising residues 182-197 of N$^{SARS\text{-}CoV\text{-}2}$ and different species of phosphorylated peptide. a, TOCSY spectrum of the non-phosphorylated peptide. **b**, TOCSY spectrum of the peptide phosphorylated only at S188. **c**, TOCSY spectrum of the twice phosphorylated peptides in a 1:1 mixture obtained from the phosphorylation reaction. **d**, Plot of the HN chemical shift perturbation (CSP) of each phosphorylated peptide (pS188 in black, pS188+pS194 in red, pS186+pS188 in blue) with respect to the non-phosphorylated one.

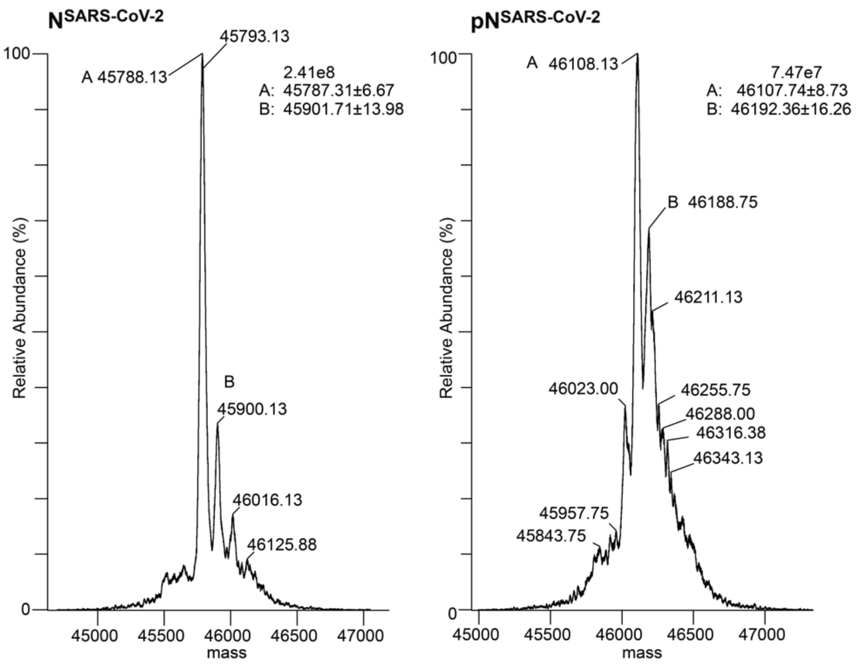

Supplementary Fig. 8. Phosphorylation of $N^{SARS\text{-}CoV\text{-}2}$ using the kinase SRPK1. Left panel, mass spectrometry of unmodified $N^{SARS\text{-}CoV\text{-}2}$, right panel mass spectrometry of SRPK1-phosphorylated $N^{SARS\text{-}CoV\text{-}2}$. The identified masses indicate 4 to 5 sites being phosphorylated by SRPK1 in $N^{SARS\text{-}CoV\text{-}2}$.

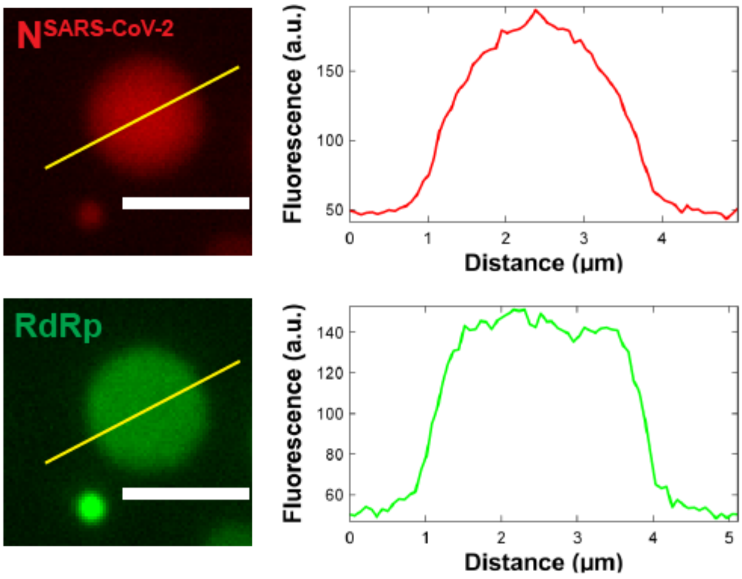

Supplementary Fig. 9. Partitioning of the RdRp/RNA-complex into droplets formed by 50 µM N$^{SARS\text{-}CoV\text{-}2}$ and 1 µM polyU. Fluorescence intensity measurement across the yellow line was plotted against the distance in micrometers. Strong enrichments of Alexa Fluor 594 fluorescently labeled N$^{SARS\text{-}CoV\text{-}2}$ and the RdRp-complex with a fluorescein-labeled minimal RNA hairpin template were observed inside the droplets in 20 mM NaPi, pH 7.5. Scale bar 3 µm. Micrographs are representative of three independent biological replicates.

Supplementary Table 1 – Primer sequences

Primer	Sequence (5'-3')
nsp12 forward	TACTTCCAATCCAATGCATCTGCTGACGCTCAGTCCTTCCTG
nsp12 reverse	TTATCCACTTCCAATGTTATTATTGCAGCACGGTGTGAGGGG
nsp8 forward	TACTTCCAATCCAATGCAGCAATTGCAAGCGAATTTAGCAGCCTG
nsp8 reverse	TTATCCACTTCCAATGTTATTACTGCAGTTTAACTGCGCTATTTGCACG
nsp7 forward	TACTTCCAATCCAATGCAAGCAAAATGTCCGATGTTAAATGCACCAGC
nsp7 reverse	TTATCCACTTCCAATGTTATTACTGCAGGGTTGCACGATTATCCAGC
