# Supplementary material for: Nucleocapsid protein of SARS-CoV-2 phase separates into RNA-rich polymerase-containing condensates
Source: Nat Commun. 2020 Nov 27;11:6041. doi: 10.1038/s41467-020-19843-1 (PMC7699647; doi:10.1038/s41467-020-19843-1)
Supplement: Supplementary file 2 — Description of Additional Supplementary Files [file 41467_2020_19843_MOESM2_ESM.pdf]

Description of Additional Supplementary Files

Supplementary Movie 1. Colocalization of $N^{SARS\text{-}CoV\text{-}2}$ and the stress granule marker G3BP1 in arsenite-induced stress granules in HeLa cells. Scale bar 10 µm**.**
