# Supplementary material for: Nucleocapsid protein of SARS-CoV-2 phase separates into RNA-rich polymerase-containing condensates
Source: Nat Commun. 2020 Nov 27;11:6041. doi: 10.1038/s41467-020-19843-1 (PMC7699647; doi:10.1038/s41467-020-19843-1)
Supplement: Supplementary file 4 — Reporting summary [file 41467_2020_19843_MOESM4_ESM.pdf]

nature research

Corresponding author(s): Markus Zweckstetter

Last updated by author(s): Oct 27, 2020

Reporting Summary

Nature Research wishes to improve the reproducibility of the work that we publish. This form provides structure for consistency and transparency in reporting. For further information on Nature Research policies, see our Editorial Policies and the Editorial Policy Checklist.

Statistics

For all statistical analyses, confirm that the following items are present in the figure legend, table legend, main text, or Methods section.

n/a	Confirmed	
[ ]	[x]	The exact sample size (*n*) for each experimental group/condition, given as a discrete number and unit of measurement
[ ]	[x]	A statement on whether measurements were taken from distinct samples or whether the same sample was measured repeatedly
[ ]	[x]	The statistical test(s) used AND whether they are one- or two-sided<br>*Only common tests should be described solely by name; describe more complex techniques in the Methods section.*
[x]	[ ]	A description of all covariates tested
[x]	[ ]	A description of any assumptions or corrections, such as tests of normality and adjustment for multiple comparisons
[ ]	[x]	A full description of the statistical parameters including central tendency (e.g. means) or other basic estimates (e.g. regression coefficient) AND variation (e.g. standard deviation) or associated estimates of uncertainty (e.g. confidence intervals)
[ ]	[x]	For null hypothesis testing, the test statistic (e.g. *F*, *t*, *r*) with confidence intervals, effect sizes, degrees of freedom and *P* value noted<br>*Give P values as exact values whenever suitable.*
[x]	[ ]	For Bayesian analysis, information on the choice of priors and Markov chain Monte Carlo settings
[x]	[ ]	For hierarchical and complex designs, identification of the appropriate level for tests and full reporting of outcomes
[x]	[ ]	Estimates of effect sizes (e.g. Cohen's *d*, Pearson's *r*), indicating how they were calculated

Our web collection on statistics for biologists contains articles on many of the points above.

Software and code

Policy information about availability of computer code

Data collection	NMR experiments were recorded using Topspin 3.5 (Bruker). DIC and Fluorescence experiments were performed on a Leica DM6B microscope. FRAP measurements were performed on a Leica TCS SP8 confocal microscope.
Data analysis	NMR spectra were processed with Topspin 3.6 (Bruker) and analysed using NMRFAM-SPARKY 1.4 software.<br>Microscopy pictures were processed and analyzed using the software FIJI 2.0.0-rc-69. Schindelin, J.; Arganda-Carreras, I. & Frise, E. et al. (2012), "Fiji: an open-source platform for biological-image analysis", Nature methods 9(7): 676-682, PMID 22743772, doi:10.1038/nmeth.2019. Plots and statistical analysis have been done using GraphPad Prism 8.0. The MD simulations were carried out in GROMACS (version 2018.3). MD trajectories analysis and figures have been carried using the PyMOL Molecular Graphics System (Version 1.8.4.0, Schrödinger, LLC).

For manuscripts utilizing custom algorithms or software that are central to the research but not yet described in published literature, software must be made available to editors and reviewers. We strongly encourage code deposition in a community repository (e.g. GitHub). See the Nature Research guidelines for submitting code & software for further information.

Data

Policy information about availability of data

All manuscripts must include a data availability statement. This statement should provide the following information, where applicable:

- Accession codes, unique identifiers, or web links for publicly available datasets
- A list of figures that have associated raw data
- A description of any restrictions on data availability

NMR assignments are available in the BMRB (code 50379). The mutation frequency from SARS-CoV-2 sequences were obtained from the database and resources of the China National Center for Bioinformation, 2019 Novel Coronavirus Resource (https://bigd.big.ac.cn/ncov?lang=en; downloaded June 7, 2020, with 42,176

genome sequences). Authors can confirm that the rest of the relevant data are included in the paper and/or its supplementary information files. Other data that support the findings of this study are available from the corresponding author upon reasonable request.

Field-specific reporting

Please select the one below that is the best fit for your research. If you are not sure, read the appropriate sections before making your selection.

- [x] Life sciences
- [ ] Behavioural & social sciences
- [ ] Ecological, evolutionary & environmental sciences

For a reference copy of the document with all sections, see nature.com/documents/nr-reporting-summary-flat.pdf

Life sciences study design

All studies must disclose on these points even when the disclosure is negative.

Sample size	All the available genome sequences (42176) in the China National Center for Bioinformation at the time of analysis (June 7th 2020) were used for the statistical analysis of N protein mutations.
Data exclusions	No data were excluded.
Replication	Measurement for the LLPS assays at the microscope were repeated at least three times by preparing new samples and using different protein batches. Turbidity measurements were performed in triplicates. Stress granule co-localization assay was repeated at least 3 times with independent samples. Molecular dynamic simulations have been repeated 5 times. FRAP measurements on droplets as well as FRAP of the stress granule have been performed 6 times on different regions of the same sample. All replications were successful.
Randomization	Randomization was not relevant to this study because we are not working with any particular population and our experiments can be repeated.
Blinding	Blinding was not relevant to this study because decision-making has no impact on the experiment and there is no risk of bias.

Reporting for specific materials, systems and methods

We require information from authors about some types of materials, experimental systems and methods used in many studies. Here, indicate whether each material, system or method listed is relevant to your study. If you are not sure if a list item applies to your research, read the appropriate section before selecting a response.

Materials & experimental systems

n/a	Involved in the study	
☐	☒	Antibodies
☐	☒	Eukaryotic cell lines
☒	☐	Palaeontology and archaeology
☒	☐	Animals and other organisms
☒	☐	Human research participants
☒	☐	Clinical data
☒	☐	Dual use research of concern

Methods

n/a	Involved in the study	
☒	☐	ChIP-seq
☒	☐	Flow cytometry
☒	☐	MRI-based neuroimaging

Antibodies

Antibodies used	Alexa Fluor 594 conjugated anti G3BP1 antibody was purchased from Santa Cruz Biotechnology (sc-365338 AF594).
Validation	G3BP1 (H-10) is a mouse monoclonal antibody raised against amino acids 158-251 mapping within an internal region of G3BP1 of human origin. G3BP1 (H-10) is recommended for detection of G3BP1 of human origin by Western Blotting (starting dilution 1:100, dilution range 1:100-1:1000), immunoprecipitation [1-2 µg per 100-500 µg of total protein (1 ml of cell lysate)], immunofluorescence (starting dilution 1:50, dilution range 1:50- 1:500), immunohistochemistry (including paraffin-embedded sections) (starting dilution 1:50, dilution range 1:50-1:500) and solid phase ELISA (starting dilution 1:30, dilution range 1:30-1:3000). Suitable for use as control antibody for G3BP1 siRNA (h): sc-75076, G3BP1 shRNA Plasmid (h): sc-75076-SH and G3BP1 shRNA (h) Lentiviral Particles: sc-75076-V. Positive Controls: A-431 whole cell lysate: sc-2201, A549 cell lysate: sc-2413 or K-562 whole cell lysate: sc-2203.

Eukaryotic cell lines

Policy information about cell lines

Cell line source(s)	HELA (ACC 57) cells were provided by DSMZ.
Authentication	Authentication was done by short tandem repeat (STR) DNA typing.

Mycoplasma contamination	Cell line tested negative for mycoplasma by DAPI, microbiological culture, RNA hybridization and PCD assays.
Commonly misidentified lines<br>(See ICLAC register)	None.
