# Supplementary figures and images for: Nucleocapsid protein of SARS-CoV-2 phase separates into RNA-rich polymerase-containing condensates
Source: Nat Commun. 2020 Nov 27;11:6041. doi: 10.1038/s41467-020-19843-1 (PMC7699647; doi:10.1038/s41467-020-19843-1)

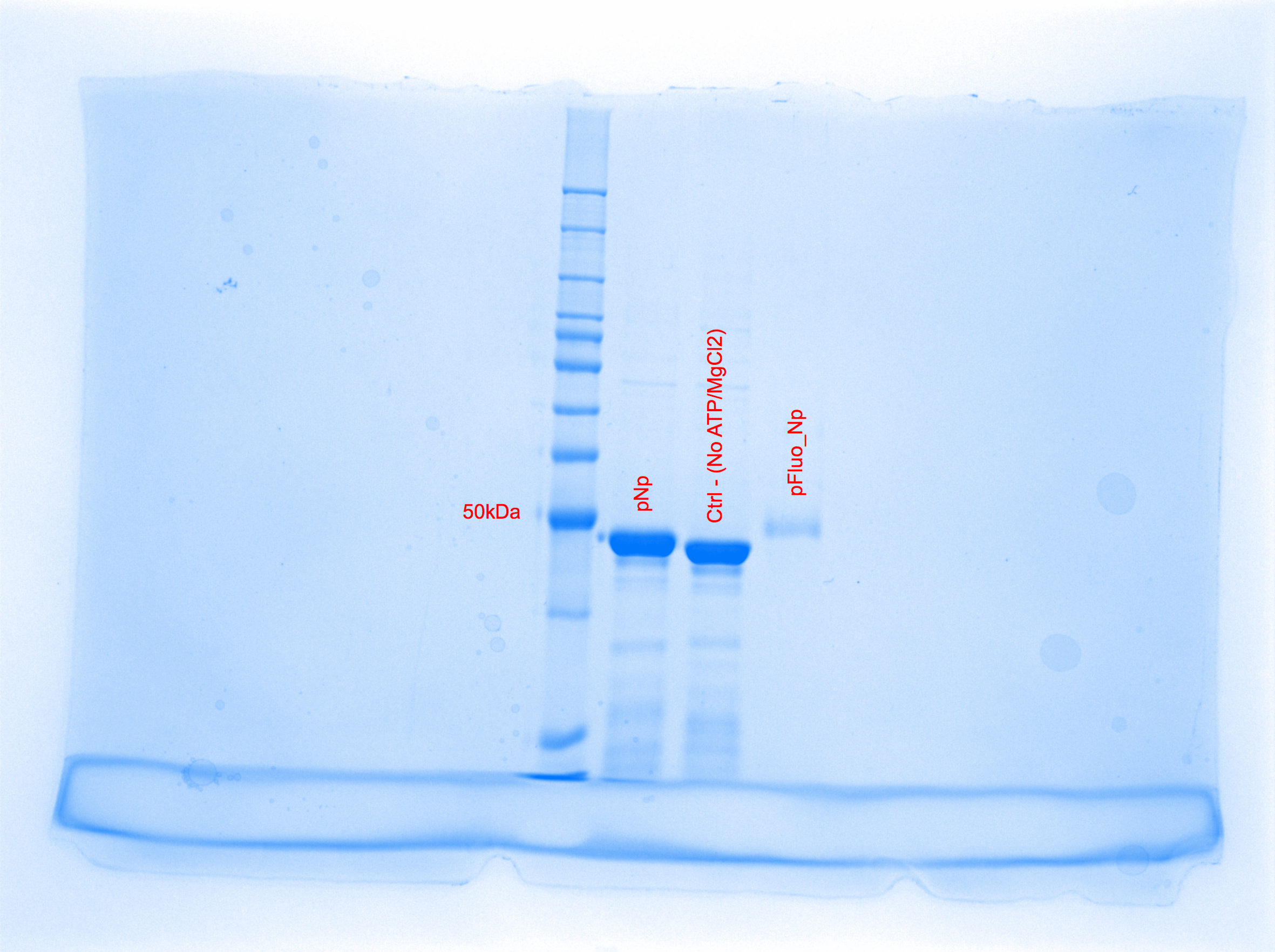

Supplement: Supplementary file 5 — Source Data [file 41467_2020_19843_MOESM5_ESM.zip › 270067_2_data_set_5041588_qzwzyt (1).tif]
